# Deep Modeling of Regulating Effects of Small Molecules on Longevity-Associated Genes

**DOI:** 10.3390/ph14100948

**Published:** 2021-09-22

**Authors:** Jiaying You, Michael Hsing, Artem Cherkasov

**Affiliations:** Vancouver Prostate Centre, Department of Urologic Sciences, Faculty of Medicine, University of British Columbia, Vancouver, BC V6H 3Z6, Canada; jyou@prostatecentre.com (J.Y.); mhsing@prostatecentre.com (M.H.)

**Keywords:** library of integrated network-based cellular signatures (LINCS), longevity, gene regulating effects, gene descriptors, molecular fingerprints, machine learning, deep neural network, drug repurposing

## Abstract

Aging is considered an inevitable process that causes deleterious effects in the functioning and appearance of cells, tissues, and organs. Recent emergence of large-scale gene expression datasets and significant advances in machine learning techniques have enabled drug repurposing efforts in promoting longevity. In this work, we further developed our previous approach—DeepCOP, a quantitative chemogenomic model that predicts gene regulating effects, and extended its application across multiple cell lines presented in LINCS to predict aging gene regulating effects induced by small molecules. As a result, a quantitative chemogenomic Deep Model was trained using gene ontology labels, molecular fingerprints, and cell line descriptors to predict gene expression responses to chemical perturbations. Other state-of-the-art machine learning approaches were also evaluated as benchmarks. Among those, the deep neural network (DNN) classifier has top-ranked known drugs with beneficial effects on aging genes, and some of these drugs were previously shown to promote longevity, illustrating the potential utility of this methodology. These results further demonstrate the capability of “hybrid” chemogenomic models, incorporating quantitative descriptors from biomarkers to capture cell specific drug–gene interactions. Such models can therefore be used for discovering drugs with desired gene regulatory effects associated with longevity.

## 1. Introduction

Aging is an ultimate, intrinsic risk factor for all degenerative conditions, and the incidence of age-associated diseases, such as Alzheimer’s, Parkinson’s, dementia, and osteoporosis (among many others), increases dramatically as we age. Moreover, humans are likely to suffer from conditions, such as vision impairment, chronic diseases, and cancers in older ages, all of which can greatly reduce the quality of life. Numerous studies were conducted in recent years to reverse the biological aging clock in animals, and a recent work has successfully demonstrated restored vision in mice by switching certain cells to a “younger” state [1]; thus, promising the possibility to regenerate tissues and organs in mammals, and encouraging researchers to explore longevity beyond laboratory animals. For example, mTOR inhibitors marked a milestone in anti-aging drug discovery and produced an FDA-approved drug, rapamycin, which extended the life spans of several model organisms. Rapamycin succeeded in increasing the lifespans by nearly three-fold in mice [2] and was proven to prolong life in yeast, worms, and flies [3]. However, there are objections to rapamycin, including warnings that such an immunosuppressive drug could lead to the development of malignancies, such as skin cancer (noted in an FDA statement). Moreover, irreversible side effects, such as diabetes [4], are also main concerns that have prevented the use of rapamycin at a larger scale. In recent years, a variety of similar studies have proposed geroprotector candidates that could potentially promote life spans [5,6,7]. For example, acarbose [8], initially used to treat diabetes, showed significant effects in improving the health and life spans of mice.

Recent developments in genomics and transcriptomics have led to a vast collection of large-scale gene expression datasets. Connectivity Map (CMap) [9], introduced in 2006, is aimed to link connections among genes, drugs, and diseases, by comparing gene signatures with reference perturbations; thus, it is a great resource when developing drug candidates with desired efficacies. CMap data have greatly been used in the bioinformatics field, especially in drug discovery applications, to retrieve novel chemicals that share similar regulatory effects on gene expressions with known perturbations. The NIH Library of Integrated Network-based Cellular Signatures (LINCS), inspired by the success of CMap, was funded as a next generation platform, using a more advanced approach at a lower cost, producing high-throughput gene expression profiles that have outpaced CMap. LINCS, with data stored in NCBI Gene Expression Omnibus (GEO), describes over 1 M gene perturbations, inflicted by thousands of small molecules at a variety of conditions and across multiple cell lines. With the increasing availability in gene expression profiles, we now have the opportunity to study how small molecules affect genes in human cells and to utilize the available gene expression data to predict drug responses, offering tremendous value for drug discovery and repurposing. For example, the limited biological knowledge on the recent COVID-19 outbreak made it difficult to choose appropriate treatments; however, querying differentially expressed genes in similar diseases (SARS-CoV-2) against CMap, to detect similarly behaved drug candidates without any prior knowledge, was shown to be an efficient therapeutic strategy [10]. In addition, rapidly emerging machine learning technologies provide powerful computational tools to discover the underlying biological mechanisms in a variety of domains. Thus, our previous study, DeepCOP, has proven the capacity of deep learning models in predicting gene expression regulating effects using LINCS perturbation datasets [11].

In this work, we propose repurposed anti-aging drug candidates by analyzing their regulation effects on the expression of pro-longevity and anti-longevity genes from the LINCS dataset. While simply querying LINCS is still a valid method to repurpose the existing drugs, this approach is limited to a very small portion of the chemical space with only about 5000 compounds. Moreover, most of the experiments described in the CMap/LINCS depository were designed to measure perturbation responses in cancer cell lines; thus, making it challenging to study longevity effects of drugs in normal, non-tumorous cell lines. Thus, it is essential to build more general machine learning models that can harness the existing data from LINCS and apply to larger chemical space and non-cancer cell lines.

Herein, we hypothesize that deep neural network (DNN) could learn from high dimensional features, including gene ontology terms, small molecule descriptors, cell line mutation, and methylation data to produce reliable predictions on drug–gene regulation effects across multiple cell lines. To build testable computational models to predict regulating effects on unknown data, we applied assorted classification approaches, including DNN, random forest (RF), Naïve Bayes, and logistic regression. We tested the drug (D)–gene (G) regulation effects on external normal cell lines using the pre-trained DNN models. We identified 13 small molecules from the LINCS dataset that demonstrated potential ability to regulate aging gene expressions with the desired effects. We further demonstrated that the efficacy of these repurposed drugs on longevity is supported by some examples from the literature.

## 2. Results and Discussion

### 2.1. Sample Distributions

We have labeled the upregulated and downregulated D–G–C interactions with the top/bottom 5% Z-score cut-off in LINCS for each cell line. This results in a comparably much smaller proportion in positive samples then the negatives. In addition, LINCS experiments are not distributed evenly across cell lines, so that the sample size differs from different cell lines. For example, cell line A375 contains 73,610 unique D–G–Cs, labeled as positive samples with top 5% threshold, while the remaining 1.2 million D–G–Cs with unknown regulating effects form the negative set. Table 1 demonstrates the unique drugs and genes in each cell line for the upregulated models.

### 2.2. CMAP LINCS Dataset Querying Results

By diving into the positive samples from the model 1 dataset labeled as upregulated D–G–C pairs, we ranked compounds that interact with the most pro-longevity genes across all LINCS cell lines. For each small molecule in the positive samples of model 1, we built a pool of (drug)–(pro-longevity gene)–(cell line) pairs and selected the molecules with the most interactions. To avoid chemicals that only upregulated pro-longevity genes within a small range of cell lines, or chemicals that only interacted with a few certain genes, we calculated the unique number of pro-longevity genes and cell lines to ensure the diversity and robustness of the selected chemicals. Only pairs that covered above 100 pro-longevity genes and more than five cell lines were included for the final ranking. Table 2 shows the top 10 small molecules that upregulate the most pro-longevity genes across all LINCS cell lines.

Conversely, positive samples in model 2 indicate downregulated D–G–C interactions for anti-longevity genes. We ranked small molecules that downregulated the most anti-longevity genes in model 2, using the same filter as Table 2, and obtained the 10 top-ranked chemicals, as shown in Table 3.

We observed seven identical drugs AT 7519, CGP-60474, trichostatin-a, alvocidib, narciclasine, oxetane, emetine in both tables, which showed not only upregulation effects with pro-longevity genes, but also downregulation effects with anti-longevity genes across multiple cancer cell lines in LINCS. In addition, PHA-793887, zibotentan, and mitoxantrone showed potential in upregulating pro-longevity gene expression, while chemical BI-2536, LSM-3353, and BMS-345541 showed downregulated expressions on anti-longevity genes. In total, we can repurpose 13 unique small molecules in LINCS perturbations for longevity purpose.

**Figure 1:**
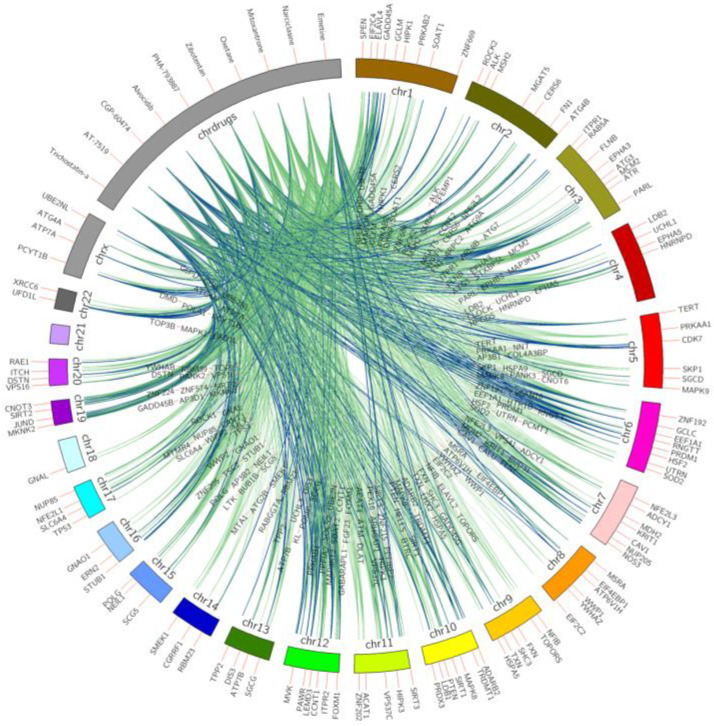
Top 10 small molecules that upregulate pro-longevity genes across all LINCS cell lines. Pro-longevity genes are shown on chromosomes bands, repurposed chemicals are shown on the drug band. Colors of interactions indicate the relationship occurrence on different cell lines. Green D–G (pro-longevity genes) links indicate the interactions were captured in less than six cell lines; pairs found in above five cell lines are labeled in blue.

**Figure 2:**
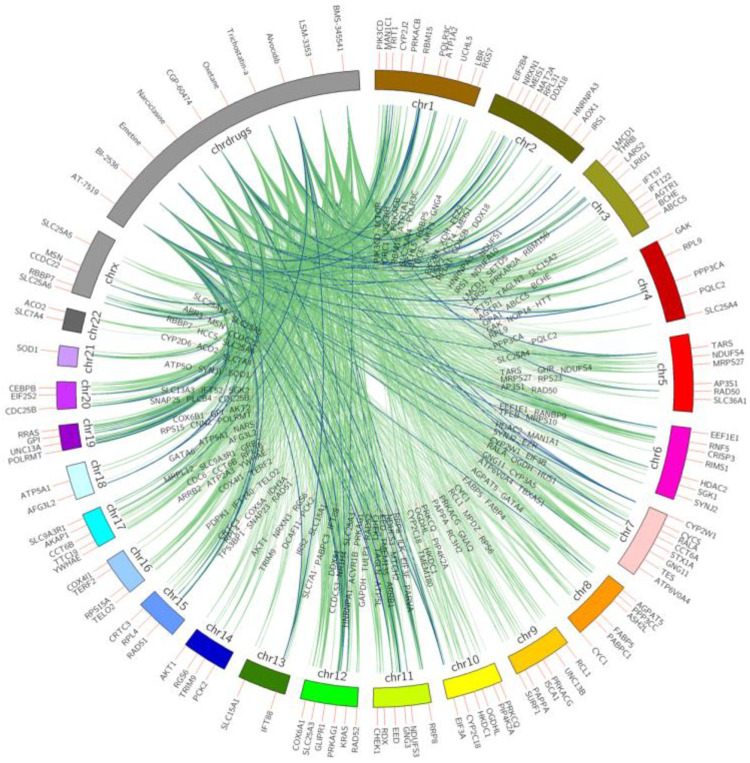
Top 10 small molecules that downregulate anti-longevity genes across all LINCS cell lines. Anti-longevity genes are shown on chromosomes bands, repurposed chemicals are shown on the drug band. Colors of interactions indicate the relationship occurrence on different cell lines. Green D–G (anti-longevity genes) links indicate the interactions were captured in less than six cell lines; pairs found in above five are labeled in blue.

Figure 1 shows the top 10 ranked small molecules with D–G (pro-longevity genes)–C interactions from model 1, and Figure 2 illustrates D–G (anti-longevity genes)–C interactions on the top 10 ranked small molecules from model 2. The color indicates the occurrence on different cell lines. From green to blue, the line connecting longevity genes with repurposed chemicals demonstrates a higher occurrence on different LINCS cell lines. For example, drug BI-2536 connects with anti-longevity gene RAD51 with downregulating effects in 9 cancer cell lines, while drug trichostatin-a promotes pro-longevity gene expressions (*GDI1*, *ZNF224*, *MAP3K13*, *EPHB1*, *ZNF500*, *PPFIA3*) in 10 cancer cell lines.

### 2.3. Model Performance

**Figure 3:**
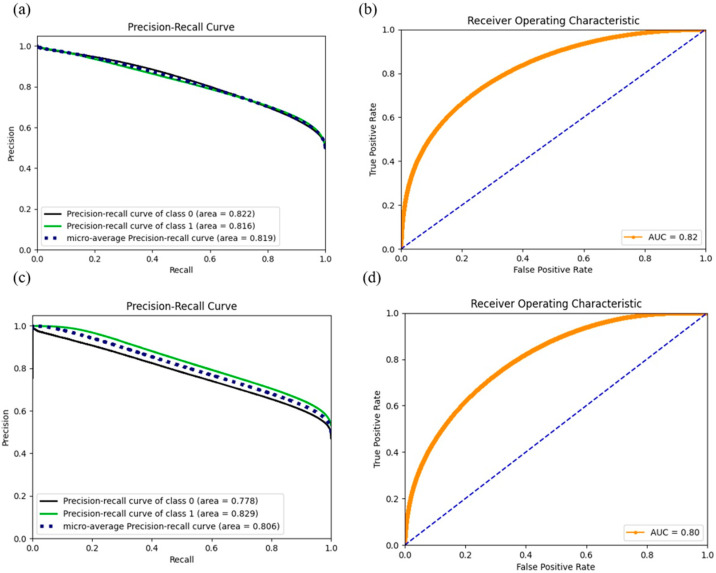
ROC and precision curves for model 1 ((**a**): PRC, (**b**): ROC) and model 2 ((**c**): PRC, (**d**): ROC). While the AUC score dropped compared with models 3–8, the dramatic increase in the APR score (positive class) gained confidence in predicting the positives in further exploration.

We estimated the accuracy parameter, AUC score, precision, and recall values for each model, as shown in Table 4. A skewed class distribution in models 3–8 failed accuracy on evaluation of the model performance. Another commonly used interpretation metric, ROC curve, was employed in binary classification problems [12] to diagnose the trade-off between sensitivity and specificity, and a higher ROC value indicates the trained model is better in distinguishing between categories. However, area under the ROC curve could be misleading when the one class significantly outweighs the other [13]. AUC score and ROC visualization could be deceptively appealing in this scenario. Instead, precision and recall provide a straightforward evaluation, focusing on the comparably small positive class based on the imbalanced dataset [13], given the concept of the precision-recall curve (PRC) being the indictor of true positives in all positive predictions. Our results demonstrate that DNN outperformed the other benchmark approaches including RF, Naïve Bayes, and ridge regression for every model, despite the selected features in terms of the APR score with an acceptable drop in AUC and accuracy. We also found that by concatenating the cell line methylation beta values and binarized mutation status, the deep neural network is more capable at extracting useful features from high-dimensional feature sets through the learning process and results in better performance than learning with single cell line annotation resource. Deep neural network models (model 1–2) have outstanding APR scores and, thus, encourage making reliable predictions in further investigations on unknown datasets. ROC and precision recall curves are shown in Figure 3 for model 1 and model 2, and curves for the rest of the DNN models are provided in the supplementary figures.

### 2.4. Prediction on Normal Cell Lines

**Figure 4:**
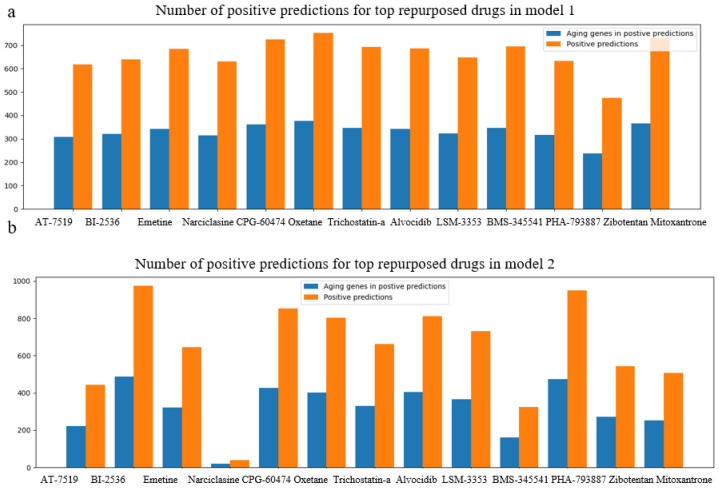
Bar charts on positive predictions for repurposed drugs in CMAP LINCS dataset, for normal cell lines, NHBEC and HGEC6B, using model 1(**a**) and model 2(**b**). Orange bars demonstrate the total number of positive predictions for each drug candidate, and blue bars illustrate the number of unique aging genes among positive predictions.

Newly generated pairs with top-ranked repurposed chemicals and longevity genes were predicted with our best-performed models—model 1 to predict upregulating effects and model 2 for downregulating the effects on normal cell lines, NHBEC and HGEC6B. In each drug candidate pool (Equation (1)), we generated D (drug candidate)–G (longevity genes)–C (normal cell line) connections as input for the pre-trained deep neural network models, and explored the positive predictions with desired regulation effects, respectively. We summarized the total positive predictions along with the number of corresponding aging genes for each drug candidate in model 1 and model 2, respectively, in Figure 4. The prediction results confirmed the efficacy of the potential desired aging gene regulation effects on normal cell lines.

(1) Overall samples of D(i)= Pool(D(i))= D(i)−G(Aging gene)−C(Normal cell line)

Table 5 shows the proportion of positive predictions against the D–G–C pool for each promising drug candidate. We observed an above 80% positive prediction rate for drugs “BI-2536”, “CGP-60474”, “oxetane”, “alvocidib” and “PHA-793887” in both model 1 and model 2, demonstrating their great potential to upregulate pro-longevity gene expression and downregulate anti-longevity gene expression in normal cells. All of the D–G–C connections in BI-2536 pool were predicted positive in model 2, meaning that ‘BI-2536′ downregulated all of the anti-longevity genes we collected from GenAge. 

### 2.5. Repurposed Drugs

**Figure 5:**
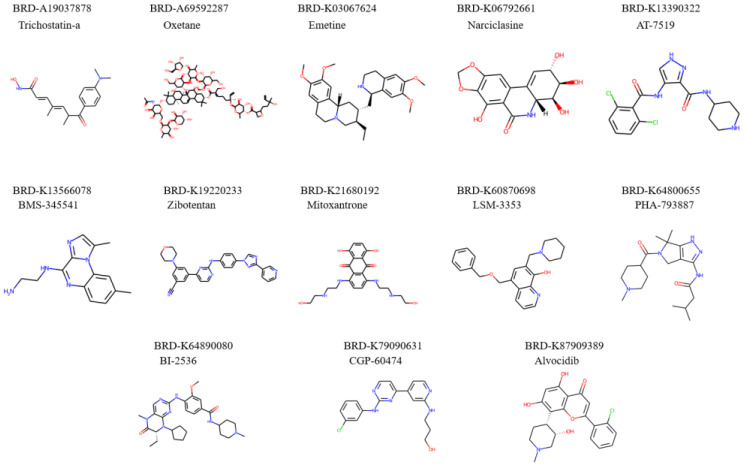
Molecular structures of repurposed drug candidates for longevity purpose.

We finally identified 13 molecules that helped to promote pro-longevity gene expressions, inhibit anti-longevity gene expressions, or act in both desired ways. Structures of repurposed molecules are shown in Figure 5. While performing experimental validations on these 13 molecules in longevity studies in model organisms is out of the scope for this paper, previous research has uncovered a number of relevant traits of those chemicals with our repurposed objectives. Table 6 summarizes the evidence that supports our findings.

Among 13 discovered longevity-promoting chemicals, four (AT 7519, alvocidib, CGP-60474, and PHA-793887) are indicated as cyclin-dependent kinase (CDK) inhibitors. Interestingly, previous studies have shown inhibition on CDK-2 resulted in tolerance towards environmental stress and promoted anti-aging in *Caenorhabditis elegans* [14,15]. Moreover, “BI-2536” inhibits tumor growth in vivo by inducing apoptosis on cancer cells as an inhibitor of polo-like kinase 1 [16]. Experiments have found the effectiveness in anti-aging on emetine dihydrochloride treated to leukemic mice [17]. In addition, narciclasine was proven to attenuate diet-induced obesity by promoting oxidative metabolism [18] while trichostatin-a, a histone deacetylase (HDAC) inhibitor, was proven to increase lifespans by promoting hsp22 gene expression on *Drosophila melanogaster* [19]. Zibotentan was designed and tested on castration-resistant prostate cancer patients as an endothelin A receptor antagonist [20], it is also proven to prevent hypertension and maintains cerebral perfusion [21]. A study conducted among 42 women with breast cancer showed great potential in mitoxantrone as a treatment for advanced breast cancer with mild side effects compared to traditional treatments, such as chemotherapy [22].

## 3. Materials and Methods

### 3.1. Datasets

Connectivity map (CMap) is a pilot project that aims to characterize cellular responses under pharmacologic perturbagens, thus, fulfilling the underdeveloped space in disease-associated gene functions, and contributing toward drug development by estimating off-target activities and eliminating unfit candidates at early stages. To date, the CMap LINCS dataset encompasses more than 1.5 million gene expression signatures related to up to 5000 small molecules and more than 10,000 genes across a total of 77 cancer cell lines [9]. Such a vast amount of gene expression information enables computational approaches, such as the deep neural network, to learn data patterns, to predict gene regulation effects [11], and drug side effects [24]. In this work, we collected L1000 high-throughput gene expression data from LINCS phase I dataset; the dataset contains perturbation data points on gene expression level under small molecular treatments at different conditions, such as dosages, cell line cultures, and time points. To reduce the data size and to maintain consistency across multiple cell lines, only perturbations with 24-h treatment were kept, and samples with molecular dose units other than “µM” were excluded.

Aging-related genes were downloaded from the GenAge (the Aging Gene Database) source, which labels pro- and anti-longevity genes in various model organisms, including (but not limited to) *Caenorhabditis elegans*, *Drosophila melanogaster*, and *Zaprionus paravittiger*. GenAge has been developed through manual curation by experts and several collaborated associations. In this work, we collected a total of 2205 genes from GenAge that are considered to have either pro- or anti-longevity effects in 10 different model organisms, including *Caenorhabditis elegans*, *Mus musculus*, *Saccharomyces cerevisiae*, *Drosophila melanogaster*, *Mesocricetus auratus*, *Podospora anserina*, *Schizosaccharomyces pombe*, *Danio rerio*, and *Caenorhabditis briggsae*. These aging-related genes identified from the model organisms were mapped to 889 human genes in total, where 397 were labeled with pro-longevity effects and 492 with anti-longevity effects, respectively (these datasets are downloadable online through https://genomics.senescence.info/download.html (accessed on 17 September 2021)). Out of a total of 889 collected aging genes, 729 were successfully mapped to the LINCS dataset and were further investigated in our models.

**Figure 6:**
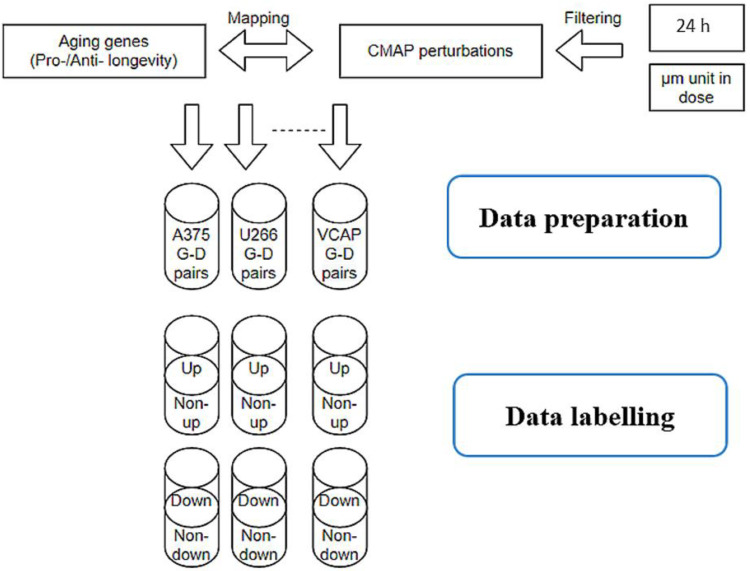
Data pipeline in collecting and labelling LINCS perturbations. Only perturbations include aging genes were kept for further analysis. The left–right percentile method was applied to label upregulation and downregulation effects with 5% threshold on the Z-score for each cell line.

**Figure 7:**
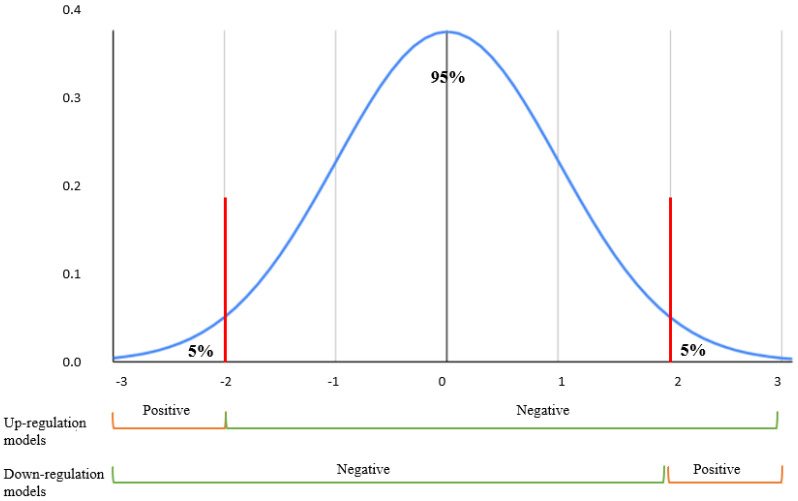
Z-score normal distribution. Positive samples were selected from the top 5% gene expressions perturbations for each cell lines in models predicting up/non-upregulation effects. Similarly, the bottom 5% perturbations were identified as the positives in models predicting down/non-downregulation effects.

Only filtered LINCS perturbations that contained 729 aging-related genes were kept for further machine learning modeling and prediction. To label gene expression regulations, we used the left–right percentile method on the Z-score with a threshold of 5% for each cell line. Only the top 5% of gene expression values were considered as upregulation samples in the upregulation models, and the bottom 5% of gene expression values were marked as downregulated in the downregulation models, while the remaining 95% samples were treated with ‘unknown’ effects (Figure 6). In the training models that predicted upregulation effects, the above defined upregulation samples were treated as positive samples, while the remaining 95% were treated as negative samples. In the training models that predicted downregulation effects, the above defined downregulation samples were treated as positive-, while the remaining 95% were treated as negative samples. Figure 7 features gene expression Z-score distribution and data sampling in upregulation and downregulation models.

### 3.2. Gene Descriptors

Gene ontology (GO) terms have been commonly used for gene annotations in recent drug discovery applications [25,26]. The GO terms consist of a set of categories that describe the gene functions as in cellular components, biological processes, and molecular functions. R package “ontologySimliarity” was built for comparing gene semantic similarity as encapsulated by the GO annotations, including nearly 20,000 terms that relate to all branches of gene ontology. The gene ontology descriptors for the 729 aging related human genes in LINCS were generated to a list of binary integers using one-hot encoding with R package “ontologySimliarity”. Only GO terms that shared with at least three age-related gene domains were selected to reduce the feature size, which resulted in 946 GO features in the final standard dataset of aging-related genes. Our previous works [11,27] already illustrated the efficacy of using GO terms as gene descriptors in machine learning models, especially with deep learning architectures.

### 3.3. Molecular Fingerprints

The molecular fingerprints were encoded into 0|1 binary vectors to encode chemical structures, where 1 indicated a specific substructure was found in a given molecule. In this paper, Morgan fingerprints [28] were generated for small molecules in the LINCS phase I dataset using the python library “RDKIT”. The Morgan fingerprints were calculated by counting the path through each atom in the chemical given a specific radius and a bit number. By increasing the radius, more fragments can be included in the Morgan fingerprint computations and can output a larger chemical feature space. We set the radius to 2 in this work and generated 2048 Morgan fingerprints for each molecular using canonical SMILES.

### 3.4. Cell Line Features

Gene mutations play an important role in cancer genetics and can be utilized to represent cell line functionalities, as previous studies have demonstrated significant performance of mutation features in machine learning approaches [29,30]. We collected copy number alternations and coding variants in “pan-cancer” from the Genomics of Drug Sensitivity in Cancer public platform, and a total of 735 mutation markers were labeled for each cell line. The mutation annotation dataset for pan-cancer is freely downloadable at https://www.cancerrxgene.org/downloads (accessed on 17 September 2021).

**Figure 8:**
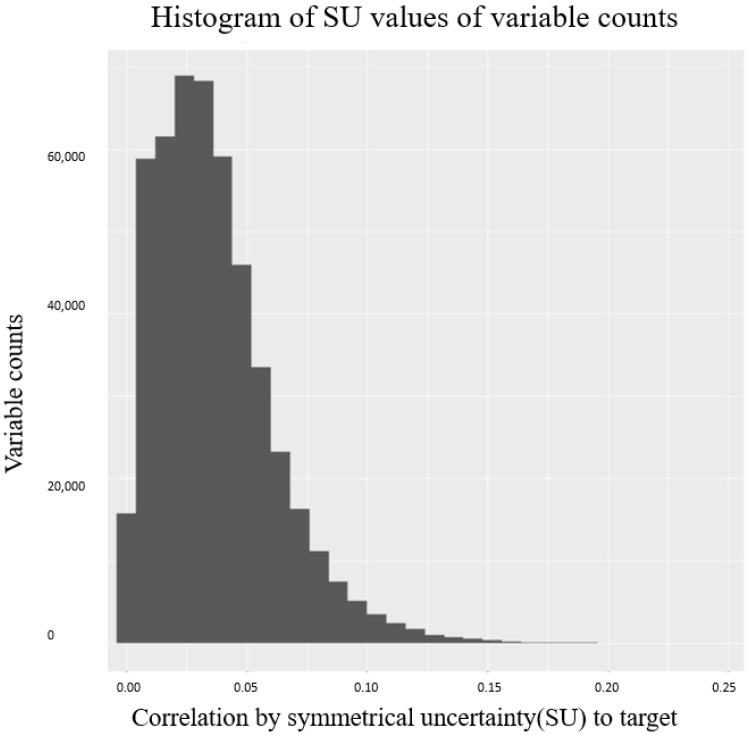
Histogram of correlation values and the corresponding variable counts using FCBF. Correlations were calculated between CpG sites and cancer histology categories.

Besides mutation markers, DNA methylation levels also contributed to drug response prediction applications [31,32]. Its impact in regulating gene expression determines organ functionalities and may cause severe diseases, such as cancer. The 450K BeadChip array provides high-throughput methylation data at more than 450K CpG sites, at a low cost, making it feasible for machine-learning algorithms to learn and extract informative features. We collected methylation profiling from the NCBI gene expression omnibus (GEO) series GSE68379, where a total of 1028 cell lines were tested with the methylation level for each CpG island. Ranging from 0 to 1, beta value was calculated as a ratio of methylated intensity verses the sum of methylated and unmethylated intensity at the probe level. The formular of beta value *B* at the specific *j* CpG site is defined as Equation (2):

(2) $$B\left(j\right)=\frac{\max\left(y_{j,methylated},0\right)}{\max\left(y_{j,methylated},0\right)+\max\left(y_{j,unmethylated},0\right)+\alpha }$$

where $y_{j,q}$ stands for $j_{th}$ probe intensities in q status. α is the added in denominator to avoid computational error. As recommended by Illumina [33], beta value is used in this work to represent the methylation level for cell lines. We used R package “FCBF” to select limited informative methylation beta values from 450 K CpG sites. The fast correlation based filter (FCBF) algorithm [34] selected the most relevant features towards histology sites of LINCS cancer cell line. Figure 8 shows the corresponding number of selected variables under a variety of (cell line)–(cancer histology sites) correlations. By choosing a correlation cut-off at 0.6, we obtained 1183 subset methylation levels for each cell line. 

### 3.5. Querying Camp LINCS Dataset

To retrieve aging-related drug (D)-gene and (G)-cell line (C) combinations, we queried perturbations with the aging-related genes in the LINCS phase I dataset. Samples were consequently labeled through 5% left–right percentiles as upregulation effects and downregulation effects, respectively. Human pro-/anti-longevity genes extracted from the GenAge platform were used as input samples to query against CMAP LINCS dataset signatures. Drugs that upregulated pro-longevity gene expression or downregulate anti-longevity gene expression across multiple cell lines were identified and could be repurposed for promoting longevity. Top chemicals, ranked by the number of D–G–C interactions, showed great potential in increasing lifespans in humans, as supported by previous studies.

### 3.6. Machine Learning Models and Deep Neural Network

Machine learning (ML) models have demonstrated unprecedented performance in recent computational biology applications [35,36,37]. ML approaches are programmed without explicit knowledge to self-extract informative features by learning the parameters, such as weights, and illustrate patterns towards the output. The pervasive applications in ML have changed our day-to-day lives, e.g., via object recognition applied in auto-driving cars, recommender systems on social media, and in-depth understanding on drug behavior. The capable solutions that trained models can learn are generally divided into regression and classification problems, where a regression model predicts the true numeric value given a set of features, and a classification model gives a category the input sample belongs. Commonly deployed classification algorithms include logistic regression, random forest (RF), and neural network (NN), each with pros and cons. It is notable that in the family of ML, deep learning (DL) plays an important part and is capable of learning more complex patterns with neurons, just as human brains. The advantage of DNN lies in absorbing datasets with high dimensions and recognizing nonlinearity; thus, providing solutions to a vast range of practical problems.

To better illustrate D–G–C relationships and have a clear evaluation on the feature power, here we differentiated the sample size and feature sets, respective to up- and downregulation effects predictions, and designed the following eight models. The selected feature set in each model is: model 1, model 2, model 3, and model 4: gene descriptor, drug descriptor, cell line mutation status, cell line methylations; model 5 and model 6: gene descriptor, drug descriptor, cell line mutation status; model 7 and model 8: gene descriptor, drug descriptor, cell line methylations.

Due to the harsh filter on the sample selection process of determining up- or down- regulation effects, only the top 5% samples were labeled as positive samples from LINCS for each model. Such severe imbalanced datasets challenged the ML approaches and could be difficult in measuring model performance for future predictions. To avoid models from being heavily influenced only by the majority class, we randomly selected the same number of negative and positive samples in model 1 and model 2. We used samples across all the cell lines in LINCS for models (1–2), and compared them with the remaining models (3–8) that used the imbalanced dataset extracted from two cell lines “U266” and “NOMO1”, which contained less data points and, thus, were easier for traditional machine learning benchmarks to train (details are provided in Table 7).

**Figure 9:**
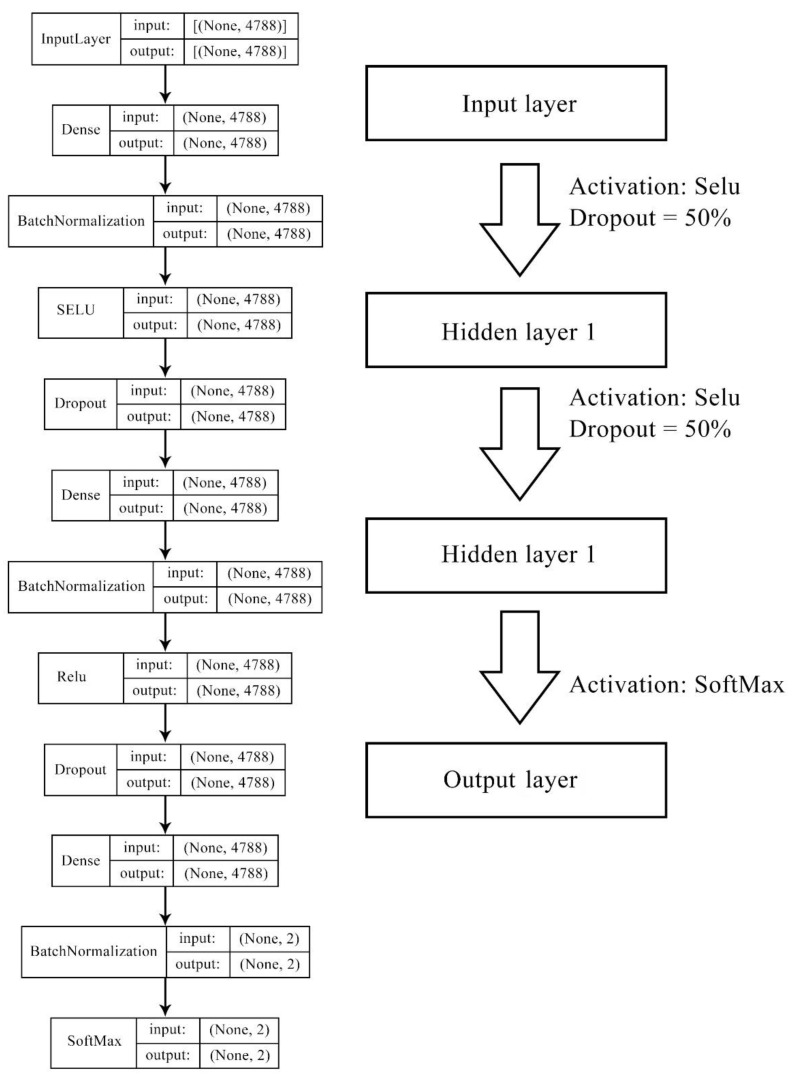
Deep neural network structure for model 1 and model 2. Features of models 1–2 were contributed by gene ontology terms (946 bits), molecular fingerprints (2048 bits), cell line mutation status (735 bits), and cell line methylation beta level (1183 bits), forming the length of 4788 bits in total.

We then compared DNN model performance with commonly used classification solving algorithms including RF, Naïve Bayes, and logistic regression. Due to the large feature size, L2 norm(ridge) regulation was applied in logistic regression models to avoid coverage failure and overfitting, by taking the squared value of trained weighs as the penalty term in the cost function. DNN was constructed with four layers (one input layer, two hidden layers, and one output layer), and the information was randomly dropped by 50% in forward propagation. Selu and Rule activation functions were used for the internal hidden layers, adding complexity and non-linearity to the model, followed by a SoftMax activation for the final output layer, to transfer values into possibilities. The neural numbers of the hidden layers were identical as those from the initial input feature list. Figure 9 illustrates the DNN structure with the number of neurons and activation functions for model 1 and model 2. For more robust evaluation results, early stopping and three-fold cross validation (CV) were applied in the DNN model to avoid overfitting. We initiated the model with hyperparameters, such as layer numbers identical with our previous studies that demonstrate decent performance, and slightly revised them by watching validation results in such a manner where model complexity must decrease when overfitting and increase when underfitting.

### 3.7. Model Evaluation

To evaluate the performance on the developed models, we computed the overall accuracy score, area under the ROC curve (AUC) parameter, as well as precision and recall values for each model. Accuracy is simply calculated as the correct prediction proportion on the whole dataset, whereas the receiver operating characteristic curve (ROC) visualizes the model performance at all classification thresholds by comparing true positive rate (TPR) versus the false positive rate (FPR). Accuracy and AUC score are commonly used in evaluating machine-learning models and offer fairly accurate insight on model performance. However, both values are easily dominated by the majority group in the imbalanced datasets and could achieve misleading high scores. To address this issue, we introduced precision and recall as supplementary evaluations. Precision (Equation (3)), also known as positive predictive value, signifies the proportion of positive samples that are predicted positive. Recall (Equation (4)), also referred as true positive rate or sensitivity, evaluates the proportion of true positives out of all predicted positive samples. Overall, precision and recall estimate the prediction power on the positives, which is highly important in our case, given that future repurposed drug candidates are based on the positive predictions and a false positive is more disastrous and costly than a false negative. As an alternative visualization of ROC curve on imbalanced datasets, the precision-recall curve (PRC) illustrates the trade-off on precision and sensitivity on every possible cut-off. A reasonable PRC curve should be above the diagonal line, with the area under the curve more than 0.5.

(3) Precision = true positive/(true positive + false positive)

(4) Recall = true positive/(true positive + false negative)

### 3.8. Prediction on Normal Cell Lines

Given the fact that all data in LINCS are for cancer cell lines, it is essential to run computational predictions for repurposing drugs with expected pro-longevity effects in normal, non-cancerous cells. The determining factor in choosing normal cell lines for prediction is the availability of features that are identical with our trained models. Two normal cell lines “NHBEC” and “HGEC6B” were tested for methylations in beta values using the same technology—Illumina 450K BeadChip arrays—and were annotated with identical CpG sites, as in GSE92843 and GSM2438425, respectively. As for the mutational status, these two normal cell lines were simply annotated as having ‘none’ in the prediction models.

**Figure 10:**
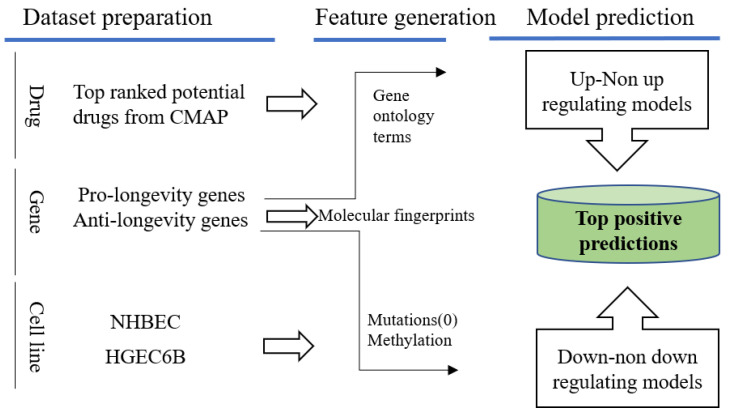
Predictions on normal cell lines with aging related genes and promising drug candidates queried from LINCS. Positive predictions will be of interest with desired regulating effects towards pro-/anti-longevity genes.

We tested the regulation effects on the top 10 ranked promising drugs that we previously queried from LINCS on these two normal cell lines, “NHBEC” and “HGEC6B”, with our best performed models, and provided the probabilities on desired regulating effects with pro-/anti-longevity genes. These 10 potential pro-longevity chemicals were paired with age genes under two normal cell lines, forming in total 7940 D–G–C pairs to be tested with up/non-upregulating (Equation (5)) and 9840 D–G–C down/non-down (Equation (6)), respectively. Figure 10 illustrates a flowchart for applying the longevity prediction models to these two normal cell lines. The mutational profiling for normal cell lines was labeled “0” in feature representation, and the methylation beta levels were collected from the Gene Expression Omnibus (GEO) “GSE92843” and “GSM2438425”.

(5) 10 (promising drugs)×397 (pro−longevity genes)×2 (cell lines)= 7940 

(6) 10 (promising drugs)×492 (anti−longevity genes)×2 (cell lines) = 9840

## 4. Conclusions

It is estimated that the anti-aging global market value was over 60 billion US dollars annually in 2020 [38]. Machine learning tools can utilize substantial transcriptional perturbation data from resources, such as CMap and LINCS, and transfer them into predictive models and actionable knowledge on modulation of longevity genes. In this study, we labeled gene expression changes using the left–right percentile at a 5% threshold for each drug–gene–cell perturbation in the LINCS datasets and analyzed the labeled samples with known human aging-related genes. We created several machine-learning models to classify the direction of gene expression changes by using combined descriptive features of small molecules and genes along with information on cell line mutations and methylation levels. The deep neural network models outperformed the other K-machine learning methods and demonstrated promising accuracy in predicting up- or down-gene-regulating effects on perturbations beyond the scope of the original LINCS dataset. In addition, we demonstrated that the longevity models, while trained from cancer cell lines, are applicable to normal cell lines, and the models predicted a list of drug candidates that could have potential to be repurposed as pro-longevity agents. Quantitative predictions on all possible combinations of D (repurposed drug)–G (aging gene)–C (normal cell line) demonstrated the desired regulating effects on normal cells for the repurposed drugs with high positive rates. As a result, we identified 13 repurposing drug candidates that could potentially promote longevity by regulating aging-related gene expressions towards the desired direction, either upregulating pro-longevity genes, downregulating anti-longevity genes, or both. Interestingly, some of the proposed drug candidates were previously reported with aging-related functionalities in a number of model organisms. For example, one of the repositioned drug candidates, trichostatin-a, was found efficient at promoting anti-aging gene expression among fruit flies [19].

Our study utilized knowledge transferring from high-throughput gene expression profiling to testable data models, and achieved accurate performance in validating regulation effects, despite the severe imbalance of the data classes. In comparison to our previous model, DeepCOP [11], which is limited to only drug and gene descriptors, our current model has incorporated additional cell line descriptors that allow knowledge to be transferred from one set of cells (for example, cancer cells) to another set of cells (such as normal cells) using a single unified DNN. This task would not have been possible with DeepCOP models, where separate, disconnected DNN models were built for each individual cell line.

The limitations of this study include the possible improved methodologies in NN schemes and the lack of experimental validation of repurposed chemicals. One noticeable NN scheme—the graph convolutional neural network (GCN)—was presented in various studies, from drug discovery [39] to gene interactions [40]. In bioinformatics applications, two major types of graph structures were applied [41]: molecular structures and interaction networks. A multi-relational interaction network could be explored in GCN models using our preprocessed dataset that contains three domains—cells, genes, and drugs. Experimental validations should also be considered in future studies, in conjunction with docking simulations and ADMET estimations (a work in progress) using our in-house drug development platforms [42].

## Figures and Tables

**Table 1:** Sample distribution for each cell line in upregulated models; positive samples are defined as the top 5% gene signatures, while negative samples are the remaining 95%. Unique numbers of drugs and aging genes are summarized below. For example, cell line A375 contains 73,610 unique D–G–Cs as positive samples with the top 5% threshold, while the remaining 1.2 million D–G–Cs with unknown regulating effects form the negatives. (#: Counts.)

Cell Lines	A375	A549	HUH7	PC3	VCAP	HL60	U266	NOMO1
Positive samples	73,610	196,027	See supplementary data	See supplementary data	See supplementary data	2138	735	1145
Negative samples	1.2M	3.4M				27,022	19,677	11,248
# Unique genes	729	729				729	729	729
# Unique drugs	1731	4875				40	28	17

**Table 2:** Top-ranked 10 small molecules that upregulate the most pro-longevity genes across all cell lines in LINCS. (#: Counts.)

Rank	Drug	# Interactions	# Unique Pro-Longevity Genes	# Unique Cell Lines
1	Trichostatin-a	926	218	10
2	AT 7519	871	226	7
3	CGP-60474	843	200	6
4	Alvocidib	743	218	6
5	PHA-793887	730	244	7
6	Emetine	714	225	6
7	Narciclasine	711	239	6
8	Zibotentan	700	218	7
9	Oxetane	673	214	6
10	Mitoxantrone	632	201	6

**Table 3:** Top-ranked 10 small molecules that downregulate the most anti-longevity genes across all cell lines in LINCS. (#: Counts.)

Rank	Drug	# Interactions	# Unique Anti-Longevity Genes	# Unique Cell Lines
1	AT-7519	585	201	7
2	BI-2536	536	207	9
3	Emetine	520	205	6
4	Narciclasine	510	212	6
5	CGP-60474	497	177	6
6	Oxetane	494	177	7
7	Trichostatin-a	454	198	6
8	Alvocidib	445	174	6
9	LSM-3353	433	215	6
10	BMS-345541	426	176	6

**Table 4:** Model performance on overall accuracy, Area under the ROC curve (AUC), and area under the precision-recall curve (PRC) for the positive class on deep neural network(DNN), random forest, naïve bayes and logistic regression models.

Model	Evaluation	DNN	Random Forest	Naïve Bayes	Logistic Regression
1	Accuracy	0.73	0.71	0.54	0.62
	AUC	0.82	0.71	0.54	0.62
	APR	0.82	0.78	0.55	0.68
2	Accuracy	0.73	0.66	0.54	0.59
	AUC	0.8	0.66	0.54	0.59
	APR	0.78	0.71	0.54	0.64
3	Accuracy	0.95	0.94	0.6	0.95
	AUC	0.89	0.64	0.76	0.63
	APR	0.46	0.39	0.12	0.44
4	Accuracy	0.95	0.95	0.59	0.95
	AUC	0.84	0.58	0.74	0.56
	APR	0.4	0.29	0.1	0.33
5	Accuracy	0.95	0.94	0.68	0.95
	AUC	0.85	0.64	0.78	0.65
	APR	0.46	0.4	0.14	0.46
6	Accuracy	0.95	0.95	0.54	0.95
	AUC	0.81	0.58	0.72	0.57
	APR	0.39	0.29	0.09	0.35
7	Accuracy	0.95	0.95	0.58	0.95
	AUC	0.88	0.64	0.75	0.64
	APR	0.46	0.4	0.11	0.45
8	Accuracy	0.95	0.95	0.58	0.95
	AUC	0.84	0.58	0.74	0.56
	APR	0.4	0.3	0.1	0.54

**Table 5:** Percentage of positively predicted D (drug candidates)–G (aging genes)–C (normal cell line) pairs for each promising drug candidate in model 1 and model 2. Highlighted repurposed drugs showed great potential in regulating aging gene expressions on normal cell lines in both models. Drugs in bold achieved high positive rate (above 80%) on both models.

Drug	Model 1 Positive Rate	Model 2 Positive Rate
AT 7519	78%	45%
**BI-2536**	81%	100%
Emetine	87%	66%
Narciclasine	80%	4%
**CGP-60474**	92%	87%
**Oxetane**	95%	82%
Trichostatin-a	88%	68%
**Alvocidib**	87%	83%
LSM-3353	82%	75%
BMS-345541	88%	33%
**PHA-793887**	80%	97%
Zibotentan	60%	56%
Mitoxantrone	92%	52%

**Table 6:** Summary of previous research findings for repurposed pro-longevity drugs.

Repurposed Drug	Traits	Evidence
AT 7519	Inhibitor of CDKs	/
BI-2536	Inhibits tumor growth	[16]
Emetine	Increases lifespan of leukemic mice	[17]
Narciclasine	Attenuates diet-induced obesity	[18]
CGP-60474	Inhibitor of CDKs	/
Oxetane	/	/
Trichostatin-a	Increases lifespan by promoting hsp22 gene expression	[19]
Alvocidib	Inhibits metastasis of human osteosarcoma cells	[23]
LSM-3353	/	/
BMS-345541	Inhibitor of kB-kinase (IKK)	/
PHA-793887	Inhibitor of pan-CDK	/
Zibotentan	Inhibits blood vessel growth	[21]
Mitoxantrone	Treatment of advanced breast cancer	[22]

**Table 7:** Detailed model layouts on predictive labels, sample cell lines, used feature set, and whether the negative class is being downsampled. For example, model 3 was trained with gene ontology term, drug descriptors, both cell line mutation status, and methylation values on perturbation responses on cell lines “U266” and “NOMO1”. The positive samples from model 3 were D–G–C interactions with the top 5% upregulated gene expression signatures, whereas the negative samples were the remaining 95% perturbation data.

Model Predictive Direction	Cultured Cell Lines	If Balanced Sample Feature
1 Up/Non-upregulation	Includes all cell lines	True, Type 1
2 Down/Non-downregulation	Includes all cell lines	True, Type 1
3 Up/Non-upregulation	U266, NOMO1	False, Type 1
4 Down/Non-downregulation	U266, NOMO1	False, Type 1
5 Up/Non-upregulation	U266, NOMO1	False, Type 2
6 Down/Non-downregulation	U266, NOMO1	False, Type 2
7 Up/Non-upregulation	U266, NOMO1	False, Type 3
8 Down/Non-downregulation	U266, NOMO1	False, Type 3

Feature definitions: Type 1: gene ontology descriptors + drug fingerprints + cell line mutational status + cell line methylation levels. Type 2: gene ontology descriptors + drug fingerprints + cell line mutational status. Type 3: gene ontology descriptors + drug fingerprints + cell line methylation levels.

## Data Availability

Data are contained within the article or Supplementary Material.

## Supplementary Materials

The following are available online at https://www.mdpi.com/article/10.3390/ph14100948/s1, Figure S1: Model 3 AUC curve, Figure S2: Model 3 PR curve, Figure S3: Model 4 AUC curve, Figure S4: Model 4 PR curve, Figure S5: Model 5 AUC curve, Figure S6: Model 5 PR curve, Figure S7: Model 6 AUC curve, Figure S8: Model 6 PR curve, Figure S9: Model 7 AUC curve. Figure S10: Model 7 PR curve, Figure S11: Model 8 AUC curve, Figure S12: Model 8 PR curve, Table S1: Sample distribution in down-regulated models, Table S2: Sample distribution in up-regulated models.

## Abbreviations

APR	area under precision recall curve
AUC	area under the roc curve
CDK	cyclin-dependent kinase
CMAP	connectivity map
CV	cross validation
FPR	false positive rate
GEO	gene expression omnibus
GO	gene ontology
LINCS	library of integrated network-based cellular signature
NN	neural networks
PRC	precision recall curve
RF	random forest
ROC	receiver operating characteristic curve
TPR	true positive rate
