# Supplementary figures and images for: Deep Modeling of Regulating Effects of Small Molecules on Longevity-Associated Genes
Source: Pharmaceuticals (Basel). 2021 Sep 22;14(10):948. doi: 10.3390/ph14100948 (PMC8539656; doi:10.3390/ph14100948)

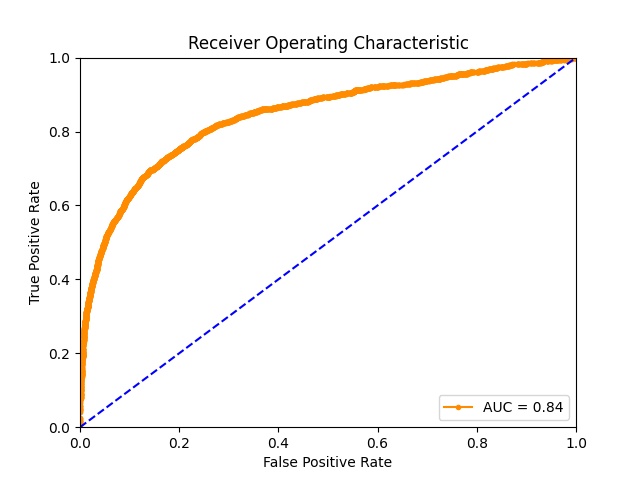

Supplement: Supplementary file 1 [file pharmaceuticals-14-00948-s001.zip › SupplementaryData/model8_auc.jpg]

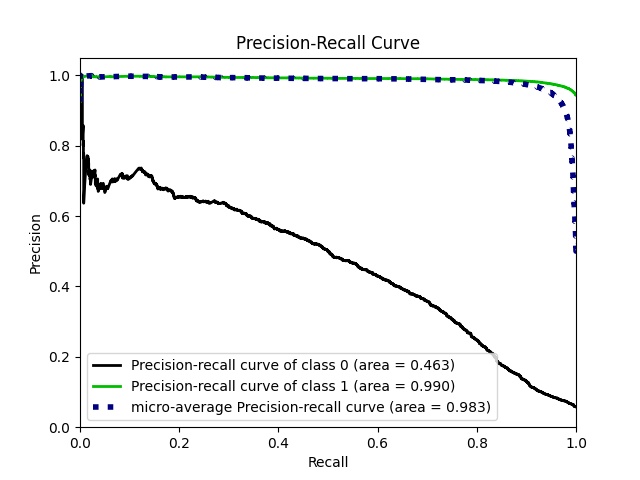

Supplement: Supplementary file 1 [file pharmaceuticals-14-00948-s001.zip › SupplementaryData/model3_pr.jpg]

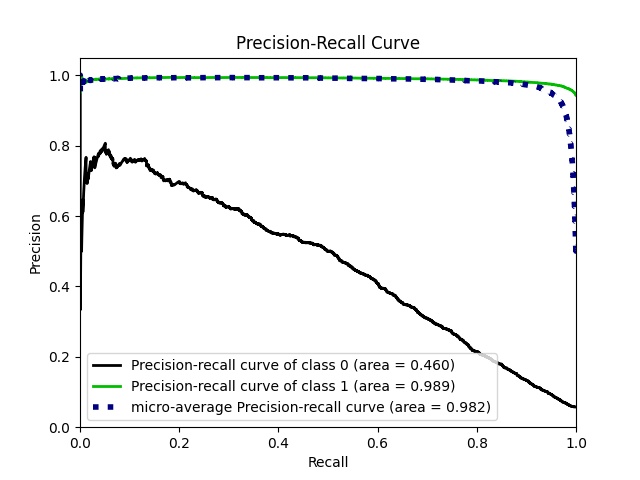

Supplement: Supplementary file 1 [file pharmaceuticals-14-00948-s001.zip › SupplementaryData/model7_pr.jpg]

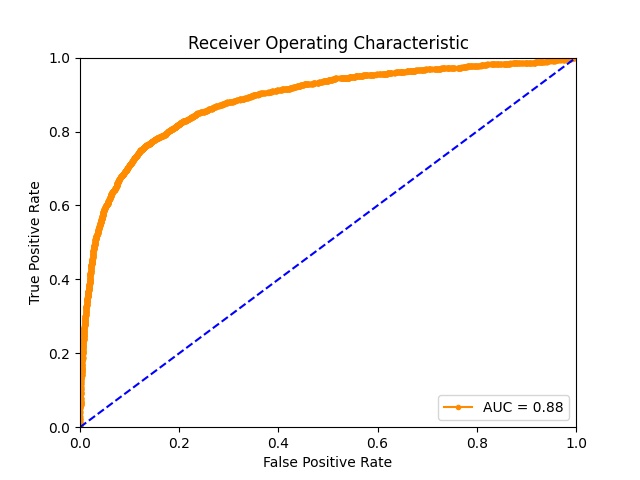

Supplement: Supplementary file 1 [file pharmaceuticals-14-00948-s001.zip › SupplementaryData/model7_auc.jpg]

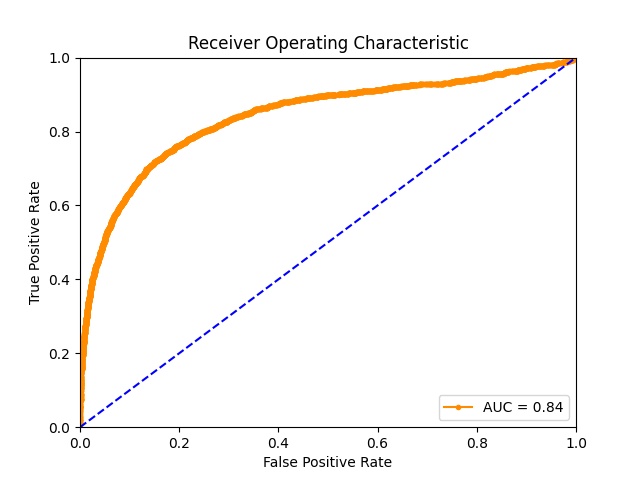

Supplement: Supplementary file 1 [file pharmaceuticals-14-00948-s001.zip › SupplementaryData/model4_auc.jpg]

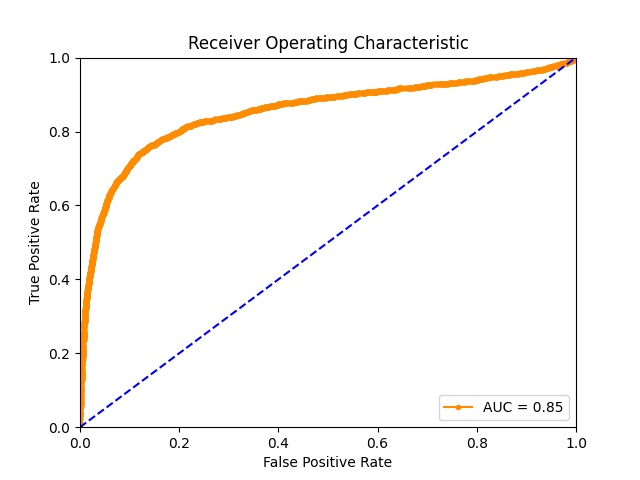

Supplement: Supplementary file 1 [file pharmaceuticals-14-00948-s001.zip › SupplementaryData/model5_auc.jpg]

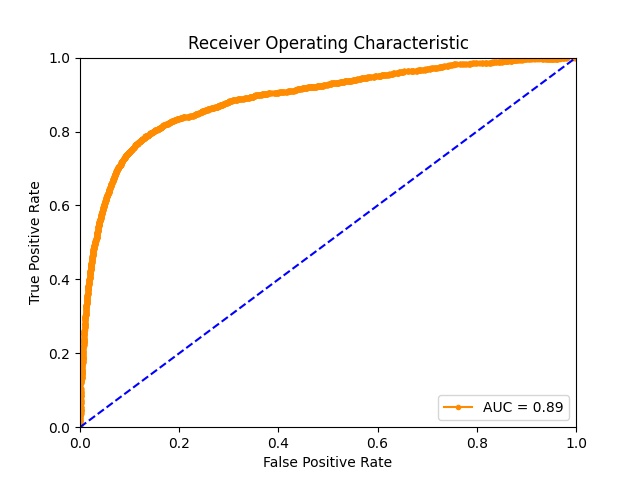

Supplement: Supplementary file 1 [file pharmaceuticals-14-00948-s001.zip › SupplementaryData/model3_auc.jpg]

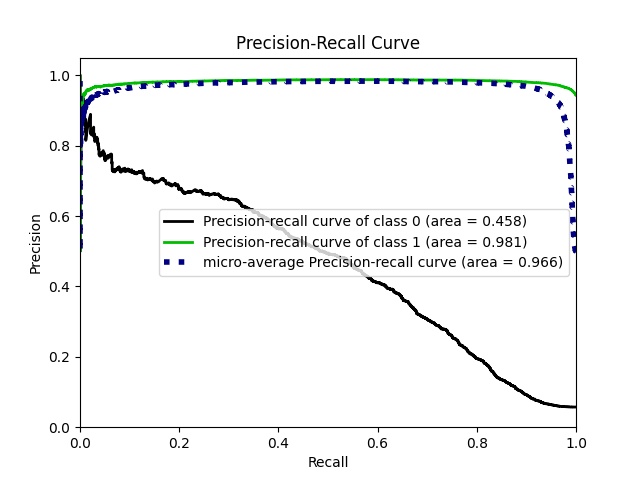

Supplement: Supplementary file 1 [file pharmaceuticals-14-00948-s001.zip › SupplementaryData/model5_pr.jpg]

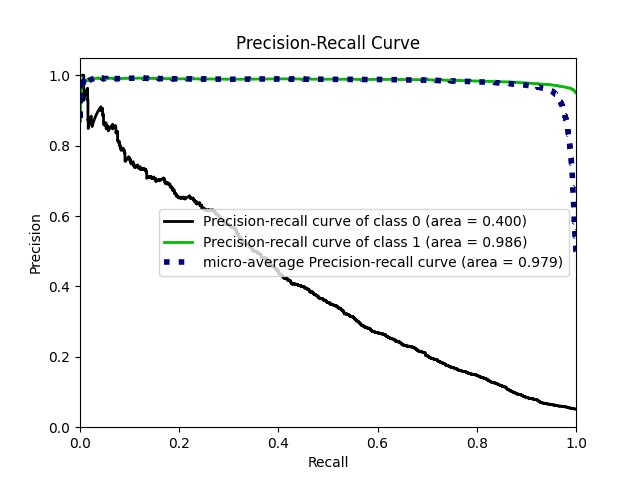

Supplement: Supplementary file 1 [file pharmaceuticals-14-00948-s001.zip › SupplementaryData/model8_pr.jpg]

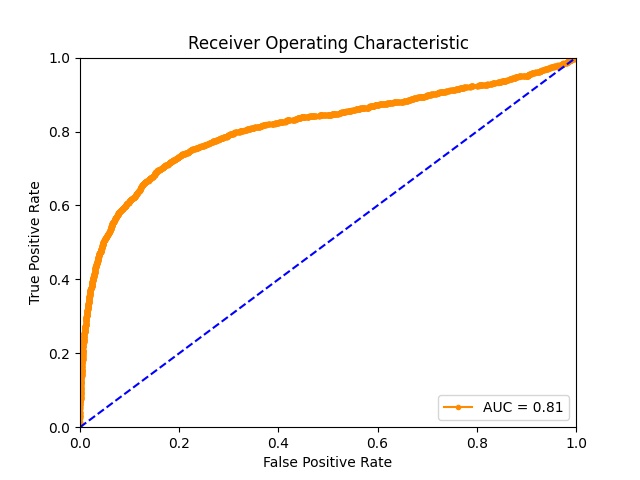

Supplement: Supplementary file 1 [file pharmaceuticals-14-00948-s001.zip › SupplementaryData/model6_auc.jpg]

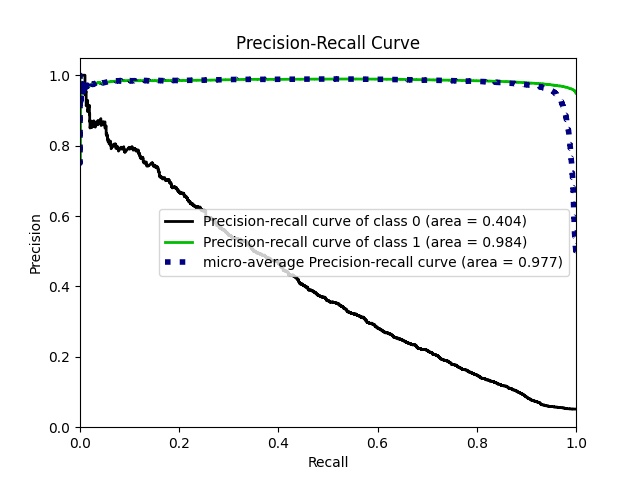

Supplement: Supplementary file 1 [file pharmaceuticals-14-00948-s001.zip › SupplementaryData/model4_pr.jpg]

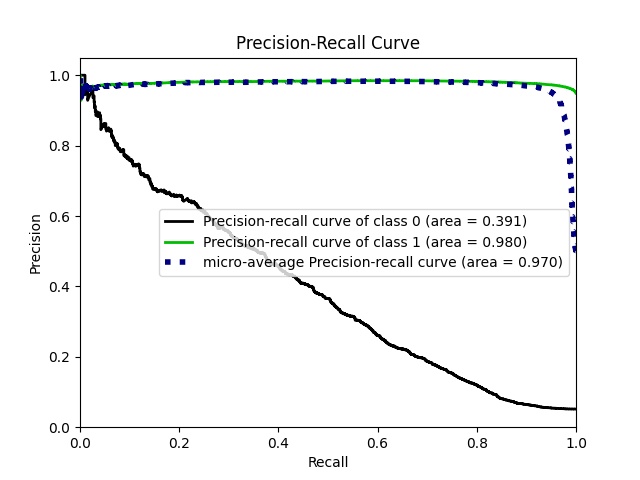

Supplement: Supplementary file 1 [file pharmaceuticals-14-00948-s001.zip › SupplementaryData/model6_pr.jpg]
